# Supplementary material for: Identification and functional analysis of non-coding regulatory small RNA FenSr3 in Bacillus amyloliquefaciens LPB-18
Source: PeerJ. 2023 May 15;11:e15236. doi: 10.7717/peerj.15236 (PMC10194069; doi:10.7717/peerj.15236)
Supplement: Supplemental Information 4 [file peerj-11-15236-s004.zip › KO/CK-vs-T1_map/map03040.html]

KEGG PATHWAY: Spliceosome - Reference pathway


|  |  |
| --- | --- |
| **Spliceosome - Reference pathway** |  |

[
Pathway menu
| Organism menu
| Pathway entry
| Show description
| User data mapping
]

After transcription, eukaryotic mRNA precursors contain protein-coding exons and noncoding introns. In the following splicing, introns are excised and exons are joined by a macromolecular complex, the spliceosome. The standard spliceosome is made up of five small nuclear ribonucleoproteins (snRNPs), U1, U2, U4, U5, and U6 snRNPs, and several spliceosome-associated proteins (SAPs). Spliceosomes are not a simple stable complex, but a dynamic family of particles that assemble on the mRNA precursor and help fold it into a conformation that allows transesterification to proceed. Various spliceosome forms (e.g. A-, B- and C-complexes) have been identified.

|  |  |  |
| --- | --- | --- |
| Reference pathway | 184% 150% 122% 100% 82% 67% 55% | 图片下载 |
